# iPSC-derived RPE as a context-of-use defined NAM framework for RPE-centered drug-induced retinal toxicity assessment

**DOI:** 10.3389/fphar.2026.1933218

**Published:** 2026-10-21

**Authors:** Weixiao Ding, Longjun Gu, Tiantian Dong, Minjun Xiong, Chen Wang, Chunlei Ji, Juan Lv, Xuefeng Zhang, Xinjiang Gong

**Affiliations:** 1 TriApex Laboratories Co., Ltd., Nanjing, Jiangsu, China; 2 Institute of Chemicobiology and Functional Materials, School of Chemistry and Chemical Engineering, Nanjing University of Science and Technology, Nanjing, Jiangsu, China; 3 School of Life Sciences and Biopharmaceuticals, Shenyang Pharmaceutical University, Shenyang, Liaoning, China

**Keywords:** context of use (COU), induced pluripotent stem cell-derived RPE (iPSC-RPE), new approach methodologies (NAMs), ocular toxicity, predictive toxicology

## Abstract

The retinal pigment epithelium (RPE) is essential for outer retinal homeostasis and a key target for drug-induced retinal toxicity. However, conventional preclinical models, including immortalized cell lines and animal systems, may incompletely recapitulate human RPE biology because of limited representation of specialized RPE functions and species-specific differences in retinal physiology. Induced pluripotent stem cell-derived RPE (iPSC-RPE) has emerged as a candidate human-relevant platform for New Approach Methodology (NAM) development, with the potential to improve mechanistic relevance by reproducing key structural and functional features of native human RPE. Its translational utility depends on a clearly defined context of use (COU), reproducible differentiation and functional characterization, and benchmark-based frameworks for future assay qualification and validation. This review evaluates the potential of iPSC-RPE as a COU-defined NAM framework for RPE-centered drug-induced retinal toxicity, with particular emphasis on toxicities in which RPE dysfunction represents a primary or mechanistically important component. We summarize differentiation strategies and critical quality attributes that may inform future assay qualification, evaluate disease-modeling evidence supporting physiological relevance, and discuss applications in ocular safety assessment, including chloroquine/hydroxychloroquine-, MEK inhibitor-, and anti-VEGF-associated retinal effects. We further discuss limitations, including cellular immaturity, donor-to-donor variability, incomplete microenvironmental modeling, and the limited validation of long-term exposure paradigms. Current evidence supports mechanistic concordance for selected RPE-centered toxicities, whereas prospective predictive performance across diverse compounds and clinically relevant exposure conditions remains to be established. Further COU-specific qualification and prospective validation will be required to establish the reliability and decision relevance of iPSC-RPE for defined ocular toxicology applications.

## 1 Introduction

The retinal pigment epithelium (RPE) is a highly polarized monolayer of pigmented epithelial cells located between the neurosensory retina and the choroid, forming the outer blood-retinal barrier (oBRB) (Schumacker et al., 2020; Subramaniam et al., 2022), and maintaining outer retinal homeostasis. Through coordinated functions including tight junction-mediated barrier integrity, directional solute transport, daily phagocytosis of photoreceptor outer segments (POS), retinoid recycling, and trophic factor secretion, the RPE is essential for photoreceptor survival and visual function (Rizzolo et al., 2011; Kaylor et al., 2017; Zhang et al., 2022). However, the same physiological complexity also renders the RPE particularly susceptible to drug-induced injury, especially under conditions that disrupt lysosomal function, mitochondrial metabolism, oxidative balance, or epithelial polarity.

From a toxicological perspective, an experimentally relevant RPE model should therefore extend beyond morphological resemblance or lineage marker expression. It should recapitulate human-relevant mechanisms underlying epithelial dysfunction, including barrier breakdown, lysosomal impairment, defective POS clearance, mitochondrial stress, and dysregulated polarized secretion. Disruption of these functions contributes to mechanisms of drug-induced retinal toxicity, which may manifest as phospholipidosis, photoreceptor degeneration, or irreversible visual impairment (Yun et al., 2018; Chia et al., 2019; Clippinger et al., 2021). Despite their widespread use, conventional preclinical models demonstrate limited predictivity for certain aspects of human ocular toxicity. Although animal models provide systemic pharmacokinetic and whole-eye context, species differences in macular architecture, photoreceptor composition, pigmentation, and blood-retinal barrier organization can limit translation of specific human retinal adverse effects. These limitations motivate the complementary use of human-relevant retinal models for mechanism-specific safety assessment (Ebeling et al., 2022; Kim et al., 2022).

Traditional cell models also present important limitations. The ARPE-19 cell line, while convenient and widely used, exhibits incomplete pigmentation, reduced epithelial polarization, low transepithelial electrical resistance (TEER) (Shi et al., 2017; Sugita et al., 2021; Maeda and Takahashi, 2023), and variable expression of key functional proteins such as RPE65, bestrophin-1, pigment epithelium-derived factor (PEDF), and vascular endothelial growth factor (VEGF) (Linetsky et al., 2020; Pfeffer and Fliesler, 2022). Primary human RPE cells (Table 1) more closely resemble native physiology but are limited by donor variability, restricted proliferative capacity, and poor scalability, which hinder their application in standardized and high-throughput screening workflows (Joachim et al., 2022; Lytvynchuk et al., 2022; Wagner et al., 2022).

**TABLE 1:** Comparative functional characteristics of ARPE-19 and mature iPSC-RPE relevant to assay qualification.

Parameter	ARPE-19	Mature iPSC-RPE	Interpretation for NAM use	References
Barrier integrity/TEER* (Ω·cm^2^)	Reported values are typically low, often <100 Ω cm^2^ under conventional culture conditions	Reported values are frequently >500 Ω cm^2^ after prolonged maturation on Transwell, although values are protocol- and laboratory-dependent	iPSC-RPE generally provides a more physiologically relevant polarized barrier; assay-specific baseline acceptance ranges should be established from historical controls rather than a universal TEER cutoff	(Fields et al., 2017; Pfeffer and Fliesler, 2022)
Polarized PEDF/VEGF* secretion	Absent, weak, or inconsistent polarization	Predominantly apical PEDF and basal VEGF secretion	Supports functional epithelial polarity and compartment-specific secretory activity	(Herardot et al., 2024)
Photoreceptor outer-segment phagocytosis	Low or incompletely physiological	Robust POS* uptake and degradation; circadian features reported in mature systems	Supports use of POS processing as a functional CQA* and mechanistic toxicity endpoint	(Yang et al., 2024; Dörschmann et al., 2025)
RPE65/Bestrophin-1 expression	Low or variable	Generally robust after functional maturation and more consistent with native RPE phenotype	Useful as identity/maturation markers, but expression alone is insufficient for functional qualification	(Johnson et al., 2017; Kiser, 2022)
Concordance with clinically relevant retinal toxicity mechanisms	Limited mechanistic fidelity for several RPE-specific toxicities	Emerging mechanistic concordance for selected toxicities, including lysosomal perturbation and MEK inhibitor-associated functional changes; prospective clinical predictivity has not been established	Supports mechanism-specific COUs*, but does not constitute validated prediction of retinal toxicity	(Urner et al., 2016; Ebeling et al., 2022; Kim et al., 2022; Lozano et al., 2026) See CQ/HCQ and MEK inhibitor studies cited in Section 2.4

* TEER, transepithelial electrical resistance; PEDF, pigment epithelium-derived factor; VEGF, vascular endothelial growth factor; POS, photoreceptor outer segments; CQA, critical quality attribute; COU, context of use. Reported TEER, values vary substantially with cell line, differentiation protocol, substrate, medium, maturation time, and measurement conditions and should therefore not be interpreted as universal thresholds for iPSC-RPE, maturity or assay qualification. “Mature iPSC-RPE” is used here descriptively to refer to preparations demonstrating functional maturation in the cited studies and should not be interpreted as a universally standardized state; maturity requirements remain dependent on the intended COU.

Although mature iPSC-RPE generally reproduce a broader range of native RPE functions than ARPE-19, this advantage is not necessarily consistent across all preparations. Functional phenotype varies according to donor/iPSC line, differentiation protocol, passage history, substrate, medium composition, culture duration, and maturation state. Therefore, iPSC-RPE should not be assumed to outperform ARPE-19 in every endpoint without COU- and assay-specific evaluation. In this context, induced pluripotent stem cell-derived RPE (iPSC-RPE) has emerged as a candidate human-relevant platform for New Approach Methodology (NAM) development because it can reproduce several key structural and functional characteristics of native human RPE (Beilmann et al., 2025; Liu et al., 2026). iPSC-RPE cells exhibit polygonal morphology, pigmentation, epithelial polarity, tight junction formation, polarized PEDF/VEGF secretion, POS phagocytosis, and partial recapitulation of retinoid cycle activity. Importantly, iPSC-RPE can reproduce selected mechanistically relevant responses to known retinal toxicants, including hydroxychloroquine-induced phospholipidosis and MEK inhibitor-associated functional alterations, supporting their utility for mechanism-based ocular safety assessment (Zushin et al., 2023; Paul et al., 2024). However, the potential regulatory utility of iPSC-RPE will depend critically on clearly defined contexts of use (COUs) and rigorous functional and predictive qualification. In regulatory science, NAM validity is not determined solely by human origin or biological complexity, but by its ability to reliably support a defined decision context with predefined performance standards, reference compounds, and measurable critical quality attributes (CQAs).

Importantly, drug-induced retinal toxicity is not restricted to the RPE. Depending on the pharmacological mechanism and exposure route, photoreceptors, Müller glia, retinal ganglion cells, vascular endothelial cells, and other retinal populations may represent primary or secondary targets. Accordingly, iPSC-RPE should not be regarded as a universal surrogate for retinal toxicity. Moreover, several forms of retinal toxicity depend on biological processes that cannot be adequately represented by an RPE monoculture. These include photoreceptor-RPE crosstalk, choroidal endothelial responses, immune-cell participation, Bruch’s membrane or extracellular-matrix interactions, and broader neurovascular coupling. Such toxicities require complementary multicellular or microphysiological systems (Vatine et al., 2019). The focus of the present review is instead on a defined COU in which RPE-centered processes, including pigmentation-dependent drug accumulation, lysosomal processing, photoreceptor outer-segment clearance, epithelial barrier function, mitochondrial homeostasis, ion/fluid transport, and polarized secretion are mechanistically relevant to RPE-associated retinal injury.

Although previous reviews have extensively addressed iPSC-RPE differentiation, disease modeling, and regenerative applications, comparatively less attention has been given to integrating RPE model qualification with NAM qualification concepts. The conceptual gap addressed in this review is the lack of a unified framework for evaluating the potential of iPSC-RPE for NAM development. Such a framework should link a defined COU to functional CQAs, mechanism-specific toxicological endpoints, reference compounds, assay performance criteria, and validation requirements. This review consequently evaluates the conditions under which iPSC-RPE may support fit-for-purpose NAM development and, equally importantly, when complementary neuronal, glial, vascular, organoid, or microphysiological models are required.

In this review, we therefore evaluate the potential of iPSC-RPE as a COU-defined NAM framework for drug-induced retinal toxicity assessment. We first summarize differentiation strategies and their impact on functional maturity and reproducibility. We then define key CQAs relevant to assay qualification, including barrier integrity, lysosomal competence, phagocytic function, mitochondrial activity, and polarized secretion. Next, we discuss disease modeling studies as mechanistic evidence supporting physiological relevance. The central section focuses on the application of iPSC-RPE in ocular safety assessment, highlighting representative toxicants and their corresponding mechanistically defined injury pathways. Finally, we critically discuss current limitations and propose a structured qualification roadmap based on CQAs, reference toxicants, inter-laboratory standardization, and exposure-relevant validation strategies.

## 2 Establishment, standardization, and COU qualification of iPSC-RPE

### 2.1 Differentiation strategies and their impact on iPSC-RPE suitability

The generation of functionally mature iPSC-RPE is a foundational requirement for their application in ocular safety assessment. However, from a regulatory toxicology perspective, differentiation is not merely a biological production step; it determines whether the resulting cell system possesses sufficient identity, stability, and functional maturity to support reproducible decision-making.

Since the establishment of human iPSC technology, RPE differentiation strategies have evolved from spontaneous pigmentation-based emergence to increasingly defined, directed, and scalable protocols (Table 2). Early spontaneous differentiation methods relied on long-term culture of iPSCs without exogenous patterning signals (Vugler et al., 2008), followed by manual isolation of pigmented epithelial-like colonies (Buchholz et al., 2013). While these approaches demonstrated intrinsic capacity of iPSCs for RPE differentiation, they are characterized by low efficiency, high batch variability, and strong dependence on cell-line-specific differentiation propensity, limiting their suitability for standardized toxicological applications. In contrast, directed differentiation strategies modulate key developmental signaling pathways, including Wnt/β-catenin, BMP, and TGF-β/Nodal signaling, to improve lineage specification and reproducibility (Leach et al., 2016; Ozaki et al., 2025). These protocols yield higher purity populations with improved epithelial morphology and functional marker expression. More recently, manufacturing-oriented approaches have been developed to meet translational and regulatory needs, including xeno-free conditions, chemically defined media, shortened differentiation timelines, and scalable monolayer-based production systems (Linkens et al., 2026; Zorin et al., 2025). These strategies are particularly relevant for iPSC-RPE deployment, where batch-to-batch consistency, inter-laboratory reproducibility, and compatibility with high-throughput screening are essential requirements.

**TABLE 2:** Representative differentiation strategies for iPSC-RPE generation.

Strategy	Subtype/Representative protocols	Key induction factors/Small molecules	Efficiency and purity	Advantages	Limitations
Spontaneous Differentiation (Buchholz et al., 2013; Leach et al., 2016; Hunt et al., 2017)	SCAC (Spontaneous Continuously Adherent Culture)	Knockout serum replacement, β-mercaptoethanol, bFGF	Low	Simplest protocol; recapitulates endogenous developmental trajectory	Very low yield and reproducibility; not all iPSC lines competent; high heterogeneity
	EB based spontaneous	Serum containing medium, no exogenous factors	Low	Mimics *in vivo* temporal sequence	Labor intensive; poorly defined; unsuitable for screening
Directed Differentiation Growth Factor Based (Sharma et al., 2022; Surendran et al., 2022)	Dual SMAD inhibition and RPE specification	SB431542 (αTGF-β), Noggin or DMH-1 (αBMP)	Moderate-high	Robust barrier formation; well characterized marker profile	Long culture period; growth factor costs; potential for EMT/de-differentiation
	BMP4/Activin A/VEGF based	BMP4, Activin A, VEGF, nicotinamide	Moderate-high	Well established; good maturation	Growth factor batch variability; high cost
	Nicotinamide enhanced	Nicotinamide, Noggin, Dkk-1, IGF-1, bFGF, Activin A, SU5402, CHIR99021	Moderate-high	Superior functional maturation; better long-term stability	Lower initial pigmentation efficiency
	Clinical grade xeno-free protocol	Sequential media: NEIM→RPEIM→RPECM →RPEGM→RPEMM; Duration: Xeno-free, feeder-free reagents	High	Xeno-free, clinically oriented production; improved translational suitability	Extended timeline; stringent reagent requirements
Directed Differentiation Small Molecule Based (Kim et al., 2010; Maruotti et al., 2015; Ito et al., 2021)	Chetomin and Nicotinamide	Chetomin (HIF inhibitor) and Nicotinamide; single passage purification	High	Small molecule only; high-throughput-compatible	Limited independent replication; functional characterization less extensive
	Tankyrase-inhibitor-based	IWR-1 endo or XAV939 (tankyrase inhibitors)	High	Overcomes line-to-line variation; resolves differentiation propensity bias	Requires subsequent CHIR99021 and SU5402 treatment for full maturation
	Triple inhibition (PD0325901 + SB431542 + Dorsomorphin) and Activin A	PD0325901 (αFGF), SB431542 (αTGF-β), Dorsomorphin (αBMP) and Activin A; B27 supplement avoided	High	Optimized marker expression; cryopreservation shortens production cycle	Requires multiple small molecules; line-specific optimization may be needed
3D Retinal Organoid Derived RPE (Linkens et al., 2026; Yang et al., 2025; Kado et al., 2026)	SFEBq (serum-free floating culture of embryoid body-like aggregates)	BMP4, Wnt antagonists (IWR-1), Matrigel	Moderate-high	Best mimics *in vivo* developmental niche; permits long-term aging studies; drusen formation modeling	Enclosed geometry; incompatible with HTS; high cost; TER difficult to measure; labor intensive
	PDMS-microwell-based 3D organoid	Defined small molecules; PDMS microwell platform for uniform organoid generation	Higher throughput	Scalable organoid production; eliminates manual microdissection	Still limited throughput for industrial screening
Chemical Reprogramming (Transdifferentiation) (Zhang et al., 2014; Deng et al., 2022)	Two-step chemical reprogramming (ciRPE)	Chemical cocktail; intermediate eye-field-like state	High	Bypasses pluripotent stage; safe and scalable; non-integrative	Very early-stage technology; limited independent validation; long-term stability not yet characterized
	Defined transcription factor mediated	CRX, MITF-A, NR2E1, C-MYC; Tet-on system for c-MYC	High	EMT resistance confers advantage for transplantation	Requires genetic modification; regulatory hurdles for clinical use

Importantly, differentiation strategy is not neutral with respect to toxicological interpretation. Protocol-related variability may influence toxicological readouts through several mechanisms. Differences in developmental patterning can alter pigmentation, epithelial polarity, barrier properties, lysosomal competence, and other functional characteristics of iPSC-RPE (Plaza et al., 2020). These differences may, in turn, affect toxicological interpretation; for example, variation in pigmentation may influence intracellular accumulation of melanin-binding compounds, differences in epithelial maturation may alter TEER and transporter expression, and variation in lysosomal or mitochondrial competence may modify the dynamic range of organelle-specific toxicity assays. Culture substrate and medium composition may further influence metabolic state and polarized secretion. Consequently, two iPSC-RPE preparations derived using different protocols may exhibit similar lineage-marker expression yet differ substantially in functional baseline and concentration-response behavior. Comparative toxicology studies should therefore report the differentiation protocol, cell line, substrate, maturation duration, baseline functional metrics, and passage or cryopreservation status. Accordingly, differentiation protocols should be selected according to the intended COU, rather than biological yield alone. For this reason, protocol harmonization should focus not only on differentiation efficiency or marker expression, but also on the reproducibility of COU-relevant functional endpoints.

For the purposes of toxicological assay development, “mature iPSC-RPE” should be defined operationally within the intended COU rather than treated as a universally standardized state or defined by culture duration alone. Depending on the intended COU, evidence of functional maturity may include convergent assessment across four domains: (i) RPE identity, including pigmentation and expression/localization of markers such as RPE65, BEST1, CRALBP, and MITF; (ii) epithelial organization, including polygonal morphology, continuous junctional proteins such as ZO-1, and stable apical-basal polarity; (iii) functional competence, including reproducible barrier function, POS uptake and degradation, lysosomal acidification, and polarized PEDF/VEGF secretion; and (iv) stability, demonstrated by maintenance of these properties over the intended assay window without substantial spontaneous EMT or loss of phenotype. No single marker, culture duration, or TEER threshold should be considered sufficient to define maturity, and the specific maturity attributes and performance expectations should be selected according to the intended COU.

An important distinction should be made between functional maturation and biological aging of iPSC-RPE. Reprogramming of somatic cells to pluripotency can reset multiple age-associated features, including epigenetic and telomere-related characteristics, thereby reducing the extent to which the biological age of the donor is retained in subsequently differentiated iPSC-derived cells (Studer et al., 2015; Rando and Chang, 2012). Consequently, even functionally mature iPSC-RPE do not necessarily reproduce the biological age of native RPE from an older individual. This limitation is particularly relevant to chronic and age-dependent retinal toxicities.

Several strategies can nevertheless be used to introduce selected aging-associated phenotypes into iPSC-derived models. General approaches include prolonged culture, chronic sublethal oxidative or mitochondrial stress, and experimentally induced aging phenotypes, including progerin-based paradigms (Miller et al., 2013). In RPE-specific models, selected features of age-associated stress can be introduced through chronic environmental or oxidative stress, perturbation of photoreceptor outer-segment processing, and exposure to aged or disease-relevant extracellular matrices (Dalvi et al., 2019; Gong et al., 2020). Photo-oxidative or lipofuscin/A2E-associated stress may further reproduce selected components of the cumulative metabolic burden experienced by native RPE. More complex co-culture or microphysiological systems may incorporate age-associated changes in extracellular matrix, vascular support, and inflammatory signaling. However, these approaches reproduce individual dimensions of aging rather than decades of physiological aging and should therefore be described as induced aging-like or aging-associated models rather than fully aged iPSC-RPE. For NAM development, the relevant aging paradigm should therefore be selected according to the intended COU and qualified using predefined age-associated functional endpoints rather than assumed to reproduce global retinal aging.

### 2.2 Functional qualification and candidate critical quality attributes (CQAs) for iPSC-RPE assay development

For iPSC-RPE to support a defined toxicological application within a fit-for-purpose NAM framework, morphological resemblance to native tissue alone is insufficient. Instead, model qualification must be defined by the extent to which the system can recapitulate human-relevant RPE functions that are mechanistically linked to drug-induced retinal toxicity. Functional qualification therefore represents the critical bridge between differentiation protocols and translational toxicology applications, determining whether a given iPSC-RPE system is merely phenotypically RPE-like or functionally competent for safety decision-making.

For clarity, four levels of evidence should be distinguished when considering iPSC-RPE for NAM development. Biological plausibility indicates that the model reproduces RPE functions implicated in retinal injury. Mechanistic relevance requires demonstration that defined perturbations produce pathway-consistent functional responses. Assay qualification requires evidence that a specified assay performs reproducibly within a defined COU, with appropriate controls, acceptance criteria, dynamic range, and reference compounds. Regulatory validation or qualification represents a higher level of evidence in which the assay has demonstrated sufficient reliability and decision relevance for an explicitly defined regulatory purpose. The framework proposed in this review primarily addresses the first three levels and should therefore be interpreted as a roadmap toward, rather than evidence of, formal regulatory qualification.

To structure this qualification process, we propose a hierarchical framework of CQAs that define iPSC-RPE qualification across different levels of biological and toxicological relevance. Candidate CQAs should satisfy four general criteria: biological relevance to the intended COU, quantitative measurability, sufficient assay dynamic range, and reproducibility across runs, batches, and cell preparations. Where the CQA is intended to support classification or decision-making, its relationship to reference-compound responses and exposure should also be demonstrated. At the most fundamental level, epithelial integrity represents a core requirement, encompassing the establishment of continuous tight junctions, stable TEER, and appropriate apical-basal polarity as reflected by proteins such as ZO-1.

In parallel, functional polarization of secretion, including directional release of PEDF apically and VEGF basally, is required to ensure that the model reproduces native RPE compartmentalization. Phagocytic competence, particularly the ability to internalize and enzymatically degrade POS, constitutes another essential functional attribute, as defective POS clearance is an important mechanism contributing to multiple forms of retinal toxicity (Brandl et al., 2014; Takagi et al., 2019). Beyond these baseline epithelial properties, mechanistic CQAs define the capacity of the model to resolve specific injury pathways. These include lysosomal acidification and autophagic flux, mitochondrial respiratory capacity and metabolic reserve, oxidative stress response systems, and the expression and functional integrity of retinoid cycle-associated proteins such as RPE65 and CRALBP. These attributes are particularly important when evaluating compounds that induce organelle-specific toxicity, metabolic dysregulation, or retinoid cycle impairment. Importantly, the expression of these markers alone is insufficient. Their functional coupling to measurable physiological outputs must be demonstrated to ensure mechanistic interpretability.

At a more exploratory level, transcriptomic, proteomic, and high-content-imaging-based phenotypic profiling can provide additional layers of biological resolution. However, while these approaches may enhance mechanistic insight and support hypothesis generation, they are not strictly required for regulatory qualification unless explicitly linked to a defined COU. Collectively, this hierarchical CQA framework enables a structured distinction between structural similarity and functional competence, thereby aligning iPSC-RPE characterization with the requirements of predictive toxicology.

Functional qualification should be distinguished from predictive validation. The CQAs proposed in this review are primarily mechanism-anchored attributes selected because they represent RPE functions linked to clinically relevant retinal injury pathways. Published studies support the responsiveness of several such endpoints to known toxicants or disease-relevant perturbations; however, such responsiveness does not by itself demonstrate prospective predictivity. At present, few iPSC-RPE assays have been tested using sufficiently large, blinded compound sets containing both clinically retinotoxic and non-retinotoxic comparators to establish sensitivity, specificity, positive predictive value, or negative predictive value. Accordingly, these CQAs should currently be regarded as candidate qualification attributes whose predictive utility remains COU dependent and requires prospective validation.

Quantitative assay characteristics are unevenly available across CQA domains. TEER is the most commonly quantified barrier metric, but reported values vary substantially with protocol, substrate, medium, maturation time, and instrumentation. POS phagocytosis is typically quantified as uptake/degradation kinetics or internalized POS per cell, but assay harmonization is limited. Lysosomal function may be quantified through lysosomal pH, cathepsin activity, autophagic-flux ratios, or POS degradation, whereas mitochondrial activity is commonly characterized by OCR/ECAR, ATP production, membrane potential, and spare respiratory capacity. Polarized secretion can be expressed as apical-to-basal PEDF/VEGF distribution or secretion ratios. However, for these endpoints, cross-study distributions, inter-laboratory coefficients of variation, and retinal-toxicity-specific sensitivity and specificity remain insufficiently characterized. Therefore, available quantitative values should presently be considered assay descriptors rather than universal qualification thresholds.

### 2.3 COU definition, functional performance boundaries, and assay acceptance criteria

A context of use should specify the toxicological question, the biological domain represented by the model, the intended decision, and the level of uncertainty acceptable for that decision. Consistent with regulatory principles for *in vitro* method development, model suitability should therefore be assessed relative to a predefined purpose rather than assumed from biological complexity alone (Poppenborg et al., 2026).

For iPSC-RPE, the COU should be stratified according to the decision being supported. Mechanistic studies require evidence that the relevant RPE pathway is faithfully represented and responsive to perturbation. Targeted hazard identification or toxicity screening additionally requires reproducible assay performance, appropriate controls, and predefined response criteria. Compound prioritization requires comparative benchmarking across positive and negative reference sets. In contrast, prediction of clinical retinal toxicity represents a substantially higher evidentiary standard and would require prospective classification performance, clinically anchored exposure-response relationships, and inter-laboratory validation. Current evidence most strongly supports the first two applications, whereas clinical prediction remains insufficiently validated. Importantly, the performance characteristics and acceptance criteria discussed here should be distinguished from experimentally validated qualification thresholds. Unless explicitly supported by existing validation data, they represent candidate considerations for future COU-specific assay development and qualification rather than established regulatory requirements.

Within a defined COU, a functional performance boundary describes the range of model characteristics within which an assay remains fit for the intended toxicological decision. These boundaries should encompass both pre-test model qualification and post-exposure assay performance. For example, a barrier-disruption COU would require a stable polarized monolayer with reproducible baseline TEER and low paracellular permeability (Fonollosa et al., 2019), whereas a lysosomal-toxicity COU would require preservation of basal lysosomal acidification and POS-processing capacity. A model failing these pre-test criteria should not be considered reliable for interpretation of the corresponding toxicity mechanism, even if cell identity markers are retained.

Assay outputs should likewise be prespecified. Barrier toxicity may be evaluated using TEER kinetics, tracer permeability, and junctional organization; lysosomal toxicity using lysosomal pH, autophagic flux, and POS degradation; mitochondrial toxicity using oxygen-consumption rate, ATP production, mitochondrial membrane potential, and recovery after washout (Floryk and Houštěk, 1999; Lin et al., 2018); and transport-related toxicity using directional fluid movement, transporter expression or activity, and reversibility. Acceptance ranges for these endpoints should be derived from historical vehicle- and reference-control distributions and subsequently refined through inter-run and inter-laboratory validation rather than imposed as universal cutoffs. Consistent with the general principles described in OECD Good *In Vitro* Method Practices (GIVIMP), assay-specific acceptance criteria can be informed by historical performance data for the test system, positive and negative controls, and analytical endpoints, and subsequently refined through validation studies (OECD, 2025). These general principles do not constitute regulatory endorsement of the specific iPSC-RPE CQAs or acceptance considerations proposed here.

For hazard classification, functional perturbation should also be interpreted relative to exposure. Benchmark concentration or concentration-response modeling can be used to identify the concentration associated with a predefined biological effect, which should then be compared with clinically relevant free-drug exposure, intraocular exposure where available, or intracellular accumulation (Cheikh et al., 2024; Hopkins et al., 2026). Because a validated retinal exposure-margin threshold has not yet been established, such margins should presently be treated as qualification variables rather than universal risk cutoffs.

Finally, predictive performance should be evaluated using independent positive and negative compounds and reported using sensitivity, specificity, positive and negative predictive values, and confidence intervals. Repeatability and reproducibility should be quantified at both the within-run and between-run levels, followed by inter-laboratory comparison. These performance characteristics define the operational boundary within which an iPSC-RPE assay could ultimately support defined toxicological decision-making and, potentially, future regulatory applications.

### 2.4 Benchmarking strategy: reference compounds, controls, and predictive validation

Benchmarking of iPSC-RPE NAMs should be considered a tiered process rather than a binary designation of an assay as validated or unvalidated. At the first level, mechanism-anchored reference compounds determine whether the model reproduces an expected biological response associated with a known retinal injury pathway. At the second level, assay-performance benchmarking establishes concentration-response behavior, temporal reproducibility, dynamic range, and discrimination between positive and negative controls. At the highest level, predictive qualification requires prospective classification of compounds with known clinical outcomes, preferably under blinded conditions and using predefined decision criteria. Most available iPSC-RPE studies currently provide mechanistic or phenotypic benchmarking, whereas systematic predictive validation remains limited (Bhattacharya et al., 2022; Fisher et al., 2022).

Chloroquine and hydroxychloroquine represent useful mechanism-anchored reference compounds for lysosome-associated retinal injury (Chandler et al., 2020). In iPSC-RPE systems, hydroxychloroquine has been used experimentally as a positive control for lysosomal alkalinization and reduced cathepsin activity (Farnoodian et al., 2022), while chloroquine has also been evaluated in iPSC-based retina-on-chip systems using lysosomal and cell-injury endpoints (Achberger et al., 2019; Gregório et al., 2021; Shang et al., 2025). Relevant readouts therefore include lysosomal pH, autophagic flux, phospholipid accumulation, POS processing, and accumulation of autofluorescent or lamellar material. However, these studies demonstrate assay responsiveness and mechanistic concordance rather than prospective predictive performance across chemically diverse compounds.

MEK inhibitors provide a mechanistically distinct benchmark because MEK inhibitor-associated retinopathy is characterized clinically by transient subretinal fluid accumulation and is frequently reversible after dose modification or withdrawal. Accordingly, functional disturbances in RPE fluid transport and homeostasis may be more informative than overt cytotoxicity. Recent preclinical studies using iPSC-RPE exposed to selumetinib have reported altered POS internalization and expression of *AQP1* and other genes related to ion and fluid transport, while epithelial membrane integrity remained comparatively preserved (Lozano et al., 2025). A subsequent patient-specific study involving two individuals further reported differential iPSC-RPE responses between a patient who developed MEKAR and one who did not (Lozano et al., 2026). These findings are mechanistically informative but remain preliminary, particularly because both studies are currently available as preprints and the patient-specific dataset is very small.

### 2.5 Regulatory precedents and translational adoption of iPSC-Based NAMs

Although iPSC-RPE assays have not yet been formally qualified by major regulatory agencies as stand-alone tools for predicting drug-induced retinal toxicity, regulatory precedents from other organ systems suggest that iPSC-derived functional models can contribute to drug-development strategies within a defined context of use. Human iPSC-derived cardiomyocyte assays provide one of the best-established examples of how iPSC-based functional models have been incorporated into safety assessment frameworks (Yang et al., 2022). Published studies and reviews have described the use of iPSC-cardiomyocyte data in regulatory submission contexts, particularly for cardiovascular safety pharmacology and mechanistic evaluation of potential liabilities (Stoelzle et al., 2026). These examples illustrate that iPSC-derived functional data may contribute to weight-of-evidence assessments rather than serve as universal replacements for conventional nonclinical studies.

Microphysiological systems provide an additional relevant precedent for the regulatory evaluation of complex human-relevant *in vitro* models. The FDA ISTAND program provides a qualification pathway for novel drug development tools within a defined context of use (COU) (Norden et al., 2026). A human Liver-Chip platform designed to evaluate drug-induced liver injury has been accepted into the ISTAND qualification pathway, representing an example of how advanced microphysiological systems may undergo structured regulatory assessment (FDA ISTAND Program; Norden et al., 2026). Importantly, entry into the ISTAND qualification process should not be interpreted as completed regulatory qualification or routine acceptance for regulatory submissions. Rather, this example illustrates the type of evidence framework, including defined COU, analytical characterization, reproducibility assessment, and demonstration of decision relevance that may be required for future qualification of advanced NAMs (Lei et al., 2026).

For iPSC-RPE, no equivalent regulatory qualification for retinal toxicity has yet been reported. Therefore, the regulatory relevance discussed in this review should be considered prospective rather than established. Lessons from iPSC-cardiomyocyte submissions and MPS qualification indicate that a successful pathway will likely require a narrowly defined COU, analytically controlled assay outputs, benchmark compounds with known clinical outcomes, predefined performance criteria, reproducibility across laboratories, and prospective demonstration of decision relevance.

## 3 Disease models as mechanistic evidence supporting iPSC-RPE NAM development

Although the primary focus of this review is the application of iPSC-RPE as a COU-defined NAM for drug-induced retinal toxicity assessment, disease modeling systems provide an important complementary layer of evidence supporting mechanistic validity. Rather than serving as therapeutic or clinical surrogates, these models demonstrate whether iPSC-RPE can faithfully recapitulate human-relevant cellular dysfunctions under defined genetic or environmental perturbations. In this sense, disease models provide mechanistic evidence supporting the biological plausibility of selected iPSC-RPE functional endpoints. Such evidence can strengthen confidence in the physiological relevance of these endpoints for safety assessment. A key advantage of iPSC-RPE disease models lies in their ability to link defined genetic alterations or controlled stressors to quantifiable epithelial phenotypes. These phenotypes often overlap with toxicity-relevant endpoints, including lysosomal dysfunction, oxidative stress, impaired phagocytosis, disrupted lipid metabolism, and barrier instability. As such, disease-associated perturbations provide a biologically grounded framework for selecting and validating CQAs used in NAM development. Importantly, however, each disease model captures only a subset of RPE biology, and therefore should not be interpreted as representing global retinal pathology.

### 3.1 Monogenic inherited retinal diseases

Monogenic inherited retinal diseases provide the most direct evidence of mechanistic fidelity in iPSC-RPE systems due to their well-defined genotype-phenotype relationships. Among these, Stargardt disease type 1 (STGD1) (Figure 1a), caused by biallelic mutations in *ABCA4*, represents a prototypical model of lysosome-centered retinal degeneration (Farnoodian et al., 2022). ABCA4 protein dysfunction leads to accumulation of bisretinoid compounds such as A2E, resulting in lipofuscin deposition, lysosomal dysfunction, oxidative stress, and progressive RPE degeneration (Marie et al., 2020; Ma et al., 2025). Patient-derived iPSC-RPE models have been shown to reproduce key pathological features, including impaired photoreceptor outer segment processing, altered lipid homeostasis, reduced lysosomal enzymatic activity, and increased autofluorescent material accumulation. These phenotypes are directly relevant to NAM development, as they overlap with mechanisms triggered by lysosomotropic drug-induced toxicity.

**FIGURE 1:**
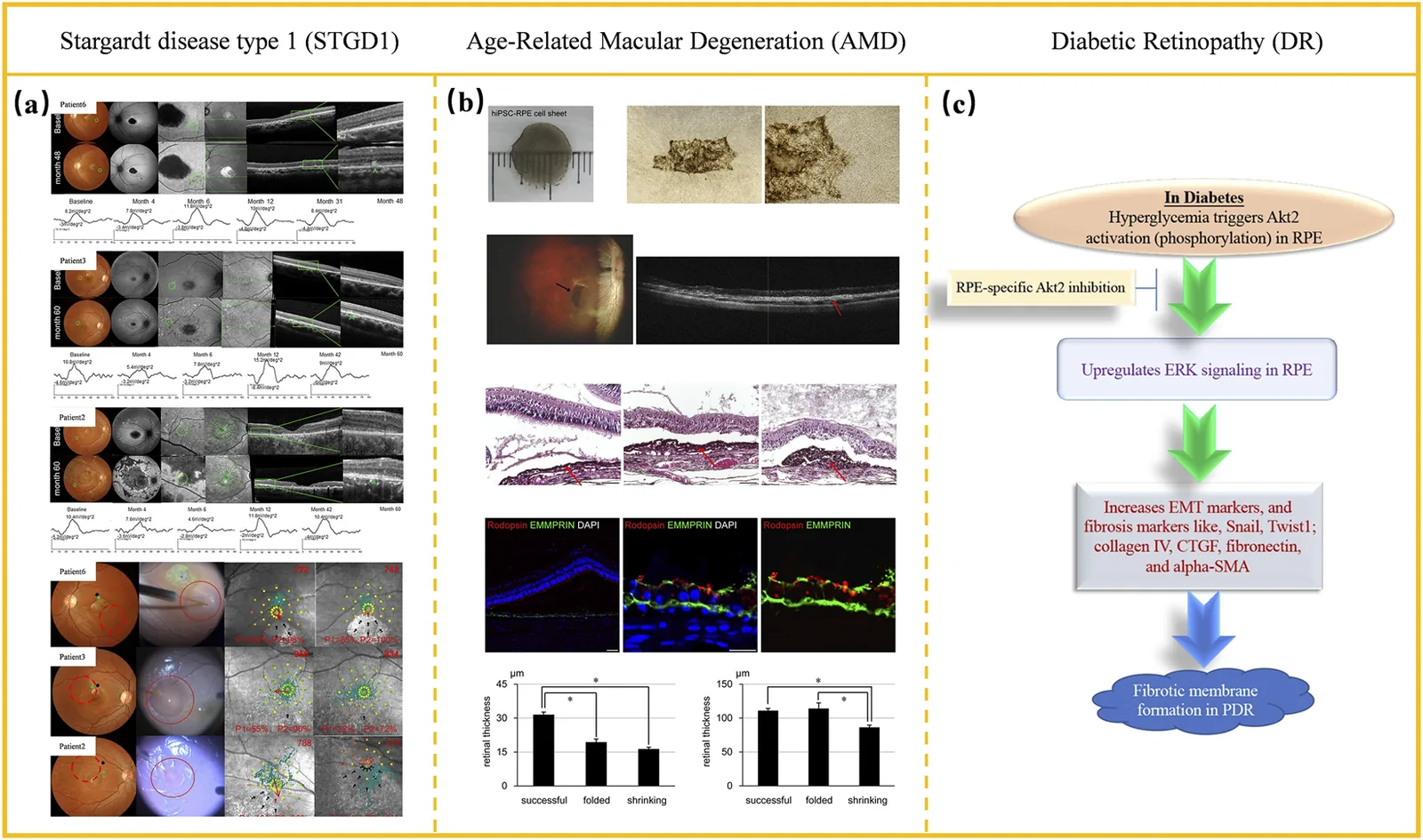
RPE-based translational models supporting mechanistic relevance of iPSC-RPE NAMs. **(a)** Human embryonic stem cell-derived RPE transplantation in early stage Stargardt macular degeneration (Li et al., 2021), illustrating the translational feasibility of stem-cell-derived RPE products in human retinal disease. **(b)** Extracellular matrix scaffold supported human iPSC-RPE cell sheet transplantation and surgical feasibility assessment (Kamao et al., 2017), highlighting the importance of epithelial polarity, matrix interaction, and tissue architecture for RPE function. **(c)** Aberrant AKT2 signaling in the RPE as a contributor to retinal fibrosis in diabetic retinopathy (Daley et al., 2023), demonstrating how RPE-centered molecular pathways can inform disease-relevant and toxicity-relevant endpoint selection.

Similarly, Best vitelliform macular dystrophy, caused by mutations in BEST1, provides a mechanistically distinct validation system centered on ion transport dysfunction (Shah et al., 2020). *BEST1* encodes a calcium-activated chloride channel localized to the basolateral membrane of RPE cells. iPSC-RPE models carrying *BEST1* mutations exhibit impaired chloride conductance, disrupted calcium signaling, altered phagocytic activity, and compromised epithelial homeostasis (Guziewicz et al., 2017; Painter et al., 2024). These defects directly connect ion transport abnormalities with functional readouts such as barrier integrity and phagocytic efficiency, both of which are critical CQAs in toxicological assessment.

Additional monogenic disorders, including *MERTK*-associated retinitis pigmentosa (Mercau et al., 2023), *TIMP3*-associated Sorsby fundus dystrophy (Hongisto et al., 2020), and EFEMP1-associated Doyne honeycomb retinal dystrophy (Rezek et al., 2026), further extend the mechanistic spectrum of iPSC-RPE models. Collectively, these systems demonstrate that iPSC-RPE can reproduce discrete, pathway-specific dysfunctions relevant to drug-induced injury (Linkens et al., 2024). These include defects in phagocytosis, extracellular-matrix remodeling, and lysosomal processing. However, each model captures only selected aspects of RPE biology and should therefore be interpreted within a pathway-specific rather than global disease context.

### 3.2 Age-related macular degeneration (AMD)

Age-related macular degeneration (AMD) (Figure 1b) is a multifactorial disease characterized by progressive RPE dysfunction (Ramkumar et al., 2010; Fleckenstein et al., 2021). Its pathogenesis involves interacting effects of genetic susceptibility, aging, oxidative stress, lipid accumulation, and complement dysregulation. Unlike monogenic diseases, AMD cannot be fully recapitulated *in vitro*; however, iPSC-RPE models provide a controlled system for dissecting individual pathogenic components that are also relevant to drug-induced toxicity mechanisms. One widely used approach involves the use of iPSC-RPE derived from donors carrying AMD-associated genetic variants, particularly complement factor H (*CFH*) polymorphisms such as Y402H (Ebeling et al., 2021). These models exhibit increased susceptibility to oxidative stress, impaired autophagic flux, lipid accumulation, and features resembling sub-RPE deposit formation (Galloway et al., 2017; Cipriani et al., 2020; Armento et al., 2024; Shang et al., 2024). Importantly, these phenotypes overlap with toxicity pathways triggered by oxidative injury and lysosomal dysfunction, thereby supporting their utility for endpoint validation in NAM frameworks.

An alternative strategy involves exposing genetically normal iPSC-RPE to AMD-relevant environmental stressors, including hydrogen peroxide, blue light, cigarette smoke extract, and lipid peroxidation products such as 4-hydroxynonenal (Lin et al., 2019; Wen et al., 2024). These perturbations induce oxidative stress, mitochondrial dysfunction, inflammatory activation, and barrier impairment, thereby modeling early-stage cellular injury rather than full disease progression. In the context of NAM development, such stress models are particularly valuable for calibrating assay sensitivity and defining threshold responses for sublethal toxicity. Complement-mediated injury models further extend this framework by introducing human serum or complement-active conditions that trigger inflammatory and degenerative changes in RPE monolayers (Fields et al., 2020; Navneet and Rohrer, 2026). While these systems remain simplified and lack full immune retinal interactions, they provide mechanistic insight into complement-driven epithelial injury and are therefore useful for evaluating complement-targeting therapeutics and inflammation-associated toxicity risk.

### 3.3 Diabetic retinopathy (DR)

Although diabetic retinopathy (DR) (Figure 1c) is traditionally considered a microvascular disease, accumulating evidence suggests that retinal pigment epithelium (RPE) dysfunction may contribute to disease progression through altered barrier integrity, trophic factor regulation, inflammatory signaling, and metabolic stress responses (Tian et al., 2026). iPSC-RPE models provide a human-relevant platform to investigate epithelial responses under diabetes-associated stress conditions, including hyperglycemia, oxidative stress, and advanced glycation-related challenges (Calzi et al., 2018). However, the ability of iPSC-RPE monocultures to fully reproduce the multicellular and vascular interactions involved in diabetic retinopathy remains an important area for further validation.

Exposure of iPSC-RPE to high glucose conditions induces oxidative stress, tight junction disruption, altered cytokine secretion, and increased apoptosis susceptibility. Although these models do not recapitulate the full neurovascular complexity of diabetic retinopathy, they are valuable for identifying how metabolic stress modifies epithelial resilience and drug response profiles. In a toxicological context, such models are particularly relevant for evaluating compounds intended for long-term administration in diabetic populations, where altered metabolic states may influence susceptibility to mitochondrial or inflammatory injury.

### 3.4 Implications for NAM development

Collectively, iPSC-RPE disease models demonstrate that the system is capable of reproducing defined, human-relevant mechanisms of retinal pigment epithelium dysfunction across genetic, degenerative, and metabolic contexts. Importantly, these models do not serve as direct surrogates for drug safety assessment; rather, they provide mechanistic anchoring points that inform the selection of relevant endpoints, refine critical quality attributes, and support the biological plausibility of observed toxicological responses (Figure 2).

**FIGURE 2:**
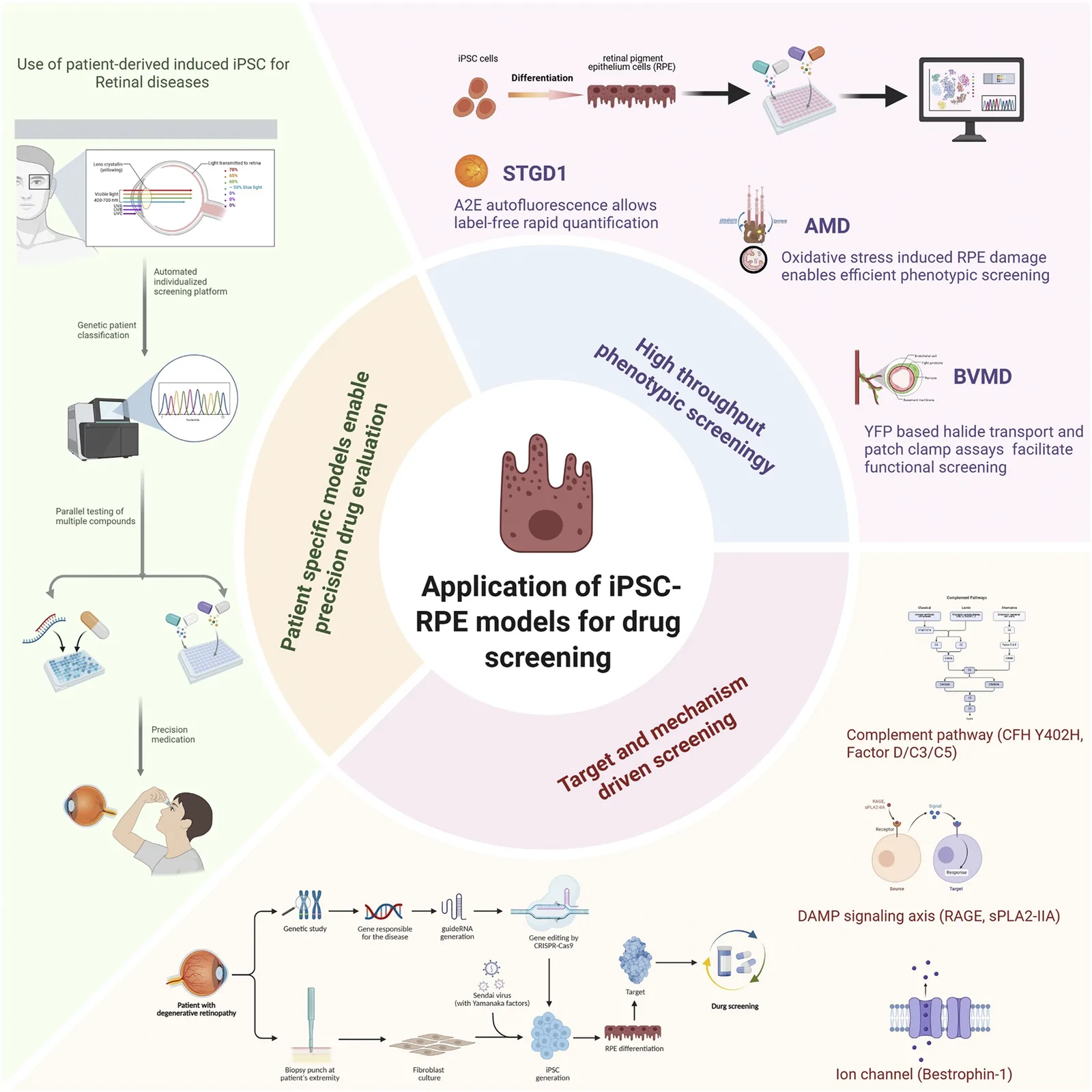
Conceptual framework for patient-specific iPSC-RPE models: from genotype-dependent phenotyping to future precision toxicology applications. Created with BioRender.com.

From a NAM perspective, the principal value of disease models lies in providing evidence that key toxicity-relevant processes, such as lysosomal degradation, oxidative-stress responses, phagocytic function, and epithelial barrier integrity, are faithfully represented in iPSC-RPE systems. This mechanistic evidence strengthens confidence in their application for COU-defined safety assessment and provides a translational bridge between cellular phenotype and clinically observed retinal injury.

## 4 Potential application of iPSC-RPE in NAM development for drug-induced retinal toxicity assessment

The primary value of iPSC-RPE in ocular safety assessment lies in its ability to translate human-relevant cellular functions into mechanistically interpretable toxicological readouts (Figure 3). Unlike conventional cytotoxicity assays that rely on terminal endpoints such as cell death, iPSC-RPE enable the detection of early functional perturbations in epithelial physiology, including lysosomal activity, barrier integrity, mitochondrial function, and polarized secretion. Importantly, these functional readouts can be mechanistically related to clinically observed patterns of retinal toxicity, supporting evaluation of mechanistic concordance between *in vitro* responses and human adverse outcomes.

**FIGURE 3:**
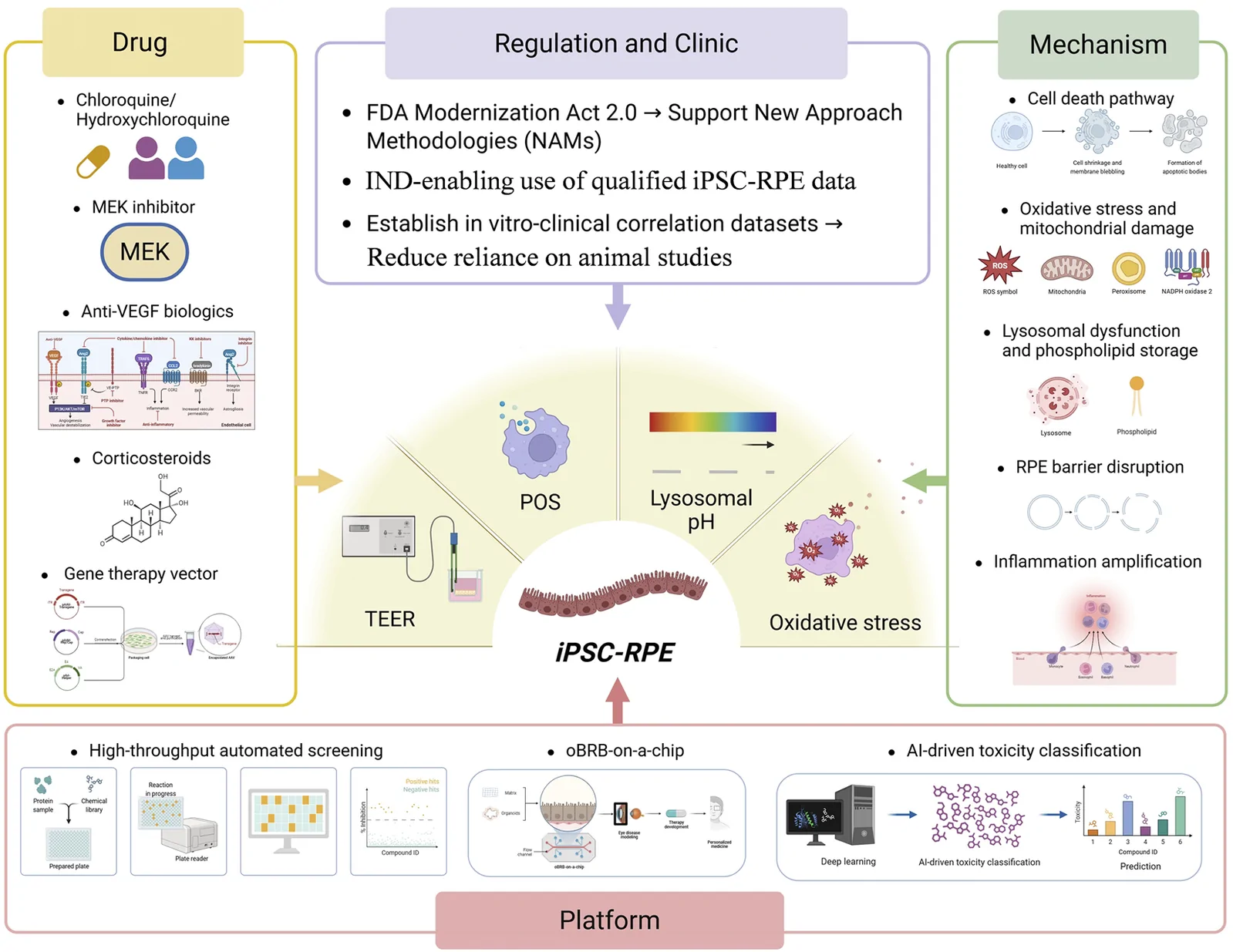
Integrated iPSC-RPE platform for ocular safety evaluation: from mechanistic dissection to translation assessment. Created with BioRender.com.

From a COU perspective, drug-induced retinal toxicity should not be treated as a single unified phenotype. Instead, different compounds may produce retinal injury through distinct, mechanism-specific pathways. Accordingly, iPSC-RPE should be considered a modular system in which individual functional endpoints are matched to specific toxicological mechanisms and COUs. In this framework, the objective of safety assessment is not only hazard identification but also mechanism resolution and toxicity pathway classification.

### 4.1 Lysosome-associated retinal toxicity: chloroquine and hydroxychloroquine

Chloroquine and hydroxychloroquine are lysosomotropic agents widely used in the treatment of autoimmune diseases such as systemic lupus erythematosus and rheumatoid arthritis (Martínez et al., 2024). Their clinical use is limited by cumulative, dose-dependent retinal toxicity characterized by irreversible photoreceptor loss and bull’s-eye maculopathy. Mechanistically, these compounds preferentially accumulate in pigmented RPE cells, where they disrupt lysosomal acidification, impair autophagic flux, induce phospholipidosis, and compromise photoreceptor outer segment degradation (Chandler et al., 2020).

iPSC-RPE systems (Brydon et al., 2019; Miyagishima et al., 2016) are particularly well suited for modeling this class of toxicity because they preserve key determinants of lysosomal function and epithelial polarity. In this context, toxicity is best understood as a progressive failure of lysosomal processing rather than acute cytotoxicity. Therefore, mechanistically informative endpoints must extend beyond viability assays and include lysosomal pH dynamics, autophagic flux markers such as LC3-II and p62, and quantitative assessment of photoreceptor outer segment degradation kinetics. These endpoints therefore provide a mechanistically grounded assay panel for lysosome-associated toxicity. However, their ability to prospectively classify chemically diverse compounds according to clinical retinotoxic potential has not yet been systematically established. In addition, intracellular autofluorescence provides a surrogate measure of lipofuscin-like accumulation, while ultrastructural analysis can confirm the presence of lamellar inclusion bodies associated with phospholipidosis.

This integrated endpoint framework enables the reconstruction of chloroquine-induced retinal injury as a multi-step lysosomal failure cascade. Importantly, it also allows comparative evaluation of structurally related compounds, thereby supporting early-stage de-risking within drug development pipelines.

### 4.2 Functional transport and reversible retinopathy: MEK inhibitors

MEK inhibitors, widely used in oncology, are associated with a distinct and often reversible ocular toxicity known as MEK inhibitor-associated retinopathy (MEKAR) (Buenasmañanas-Maeso et al., 2022). Clinically, this condition is characterized by transient serous retinal detachment and visual disturbances that typically resolve upon dose reduction or discontinuation. Unlike lysosomal toxicants, MEK inhibitors do not generally induce overt RPE cell death, suggesting that their primary effects involve functional dysregulation rather than structural degeneration.

These observations provide comparatively strong mechanistic support for transport- and fluid-homeostasis-related endpoints in the MEK inhibitor COU because the *in vitro* phenotype is consistent with the reversible fluid accumulation (Urner et al., 2016) characteristic of MEK inhibitor-associated retinopathy. Nevertheless, broader predictive validation across additional MEK inhibitors, negative comparators, and clinically relevant exposure ranges remains necessary.

Accordingly, an iPSC-RPE COU for MEK inhibitor-associated retinal effects should emphasize endpoints related to epithelial transport physiology. Relevant readouts include aquaporin-mediated fluid transport, lysosomal acidification status, ion channel and transporter expression profiles, and recovery kinetics following drug withdrawal. Importantly, reversibility after washout should be considered a defining feature of this toxicity class, emphasizing the need for dynamic rather than static experimental designs.

### 4.3 Anti-VEGF exposure: an emerging application for long-term homeostatic assessment

Anti-VEGF biologics are first-line therapies for neovascular age-related macular degeneration and other retinal vascular diseases. Although their short-term safety profile is well established, concerns remain regarding potential long-term effects of chronic VEGF suppression on retinal homeostasis, particularly given the role of RPE-derived VEGF in maintaining choriocapillaris integrity and outer retinal function. Unlike chloroquine or MEK inhibitors, anti-VEGF-related effects are not yet fully characterized within iPSC-RPE systems and should therefore be considered an emerging COU rather than a validated toxicological application (Marneros et al., 2005; Saint et al., 2009). The potential value of iPSC-RPE in this setting lies in its ability to investigate long-term perturbations of epithelial signaling networks under sustained exposure conditions.

Within this framework, relevant endpoints include polarized VEGF and PEDF secretion balance, epithelial barrier stability, mitochondrial metabolic capacity, and inflammatory or complement-associated signaling. Given the chronic nature of exposure in clinical settings, conventional short-term assays may not adequately capture cumulative or delayed functional effects. Future studies should therefore consider long-term culture systems, repeated-dosing paradigms, and recovery-based designs. Integration with endothelial co-culture or oBRB-on-chip systems may further enhance physiological relevance by incorporating vascular epithelial interactions (Skottman et al., 2017; Achberger et al., 2019; Kado et al., 2026).

### 4.4 Boundaries of iPSC-RPE COUs for other clinically recognized retinal toxicants

The relatively narrow toxicant spectrum currently available for iPSC-RPE COU development reflects the strength and mechanistic resolution of the existing evidence rather than the broader clinical spectrum of drug-induced retinal toxicity. Importantly, a clinically recognized retinal toxicant is not necessarily an appropriate reference compound for an RPE-centered NAM. For example, vigabatrin-associated retinal toxicity has been shown to be partially mediated by signaling in rod and cone photoreceptors, indicating that an RPE monoculture alone would incompletely represent the relevant biological domain (Heim and Gidal, 2012; Yang et al., 2012). Tamoxifen retinopathy is characterized by heterogeneous retinal findings, including crystalline deposits, cystoid macular edema, hyperreflective deposits in the inner retinal layers, and telangiectasia (Tenney et al., 2024). This heterogeneous phenotype makes it difficult to define a single RPE-centered mechanism and corresponding iPSC-RPE COU on the basis of clinical findings alone. Conversely, pentosan polysulfate-associated maculopathy shows prominent RPE-level abnormalities, including pigmentary and autofluorescence changes, and may therefore represent a relevant candidate for future iPSC-RPE investigation. However, its underlying pathogenic mechanism remains incompletely understood, limiting its current use as a mechanism-anchored reference toxicant for iPSC-RPE COU qualification (Hall et al., 2025).

Accordingly, other clinically recognized retinal toxicants should not be excluded from future iPSC-RPE evaluation. However, their suitability as reference compounds should be determined by evidence linking the clinical phenotype to a primary or mechanistically important RPE process, a measurable iPSC-RPE endpoint, and an exposure-response relationship. The emphasis on CQ/HCQ and MEK inhibitors in the present review therefore reflects their comparatively clearer linkage between clinically observed retinal effects, RPE-relevant mechanisms, and experimentally measurable functional endpoints, rather than an assumption that these compounds encompass the full spectrum of drug-induced retinal toxicity.

### 4.5 Mechanistic toxicity pathway mapping and NAM integration

Across different drug classes, a consistent principle emerges: retinal toxicity is mechanistically heterogeneous. It therefore cannot be adequately characterized using a single readout such as viability or morphology. Instead, toxicological phenotypes may arise from perturbations in interconnected biological systems, including lysosomal degradation, mitochondrial metabolism, epithelial transport, inflammatory signaling, and barrier integrity (Blasiak et al., 2022; Ghosh et al., 2024; Tsikandelova et al., 2024). Within a NAM framework, these mechanisms can be systematically mapped to functional assay domains. Lysosomal dysfunction is primarily captured through autophagic flux and degradation kinetics; mitochondrial toxicity through respiratory capacity and oxidative stress markers; barrier disruption through TEER dynamics and junctional integrity; and transport dysregulation through fluid flux and ion channel activity (Regent et al., 2019). This mechanistic mapping supports the transition of iPSC-RPE from a descriptive cellular model toward a structured, mechanism-informed framework for ocular safety assessment.

An additional consideration is the alignment between *in vitro* exposure conditions and clinically relevant retinal exposure. Nominal concentrations applied in cell culture do not necessarily represent free-drug, intracellular, or tissue-level exposure in the human retina because ocular barriers, protein binding, tissue distribution, transport, and clearance influence drug disposition (Dumouchel et al., 2018; Williamson and Pilla Reddy, 2021). Exposure interpretation should therefore be incorporated into the qualification strategy rather than treated as a simple adjustment of dosing concentration. Where feasible, studies should report unbound drug concentration, exposure duration, repeated-dose schedules, intracellular accumulation, and recovery following washout. These parameters are particularly important when establishing COU-specific assay performance criteria for compounds with intracellular sequestration or delayed effects (Table 3).

**TABLE 3:** Proposed COU-specific qualification framework for iPSC-RPE NAM development.

COU	Required model CQAs	Primary assay readouts	Candidate positive/reference controls	Negative/comparator controls	Proposed considerations for future assay performance and acceptance criteria	Current evidence and predictive-validation status
Lysosome-associated toxicity/phospholipidosis	Adequate pigmentation; preserved basal lysosomal acidity; competent POS uptake/degradation; stable RPE identity and polarity	Lysosomal pH; LC3-II/p62 or other autophagic-flux measures; cathepsin activity; POS degradation kinetics; autofluorescent material; EM-detectable lamellar inclusions	Chloroquine; hydroxychloroquine	Vehicle/untreated control; ideally physicochemically or mechanistically matched compounds without established retinal toxicity	For future qualification, pre-test lysosomal function could be evaluated relative to assay-specific historical control ranges. Reference compounds would be expected to demonstrate reproducible, concentration- and time-dependent lysosomal perturbation without reliance on nonspecific acute cytotoxicity. Effect thresholds and classification performance would require establishment using blinded positive/negative compound panels and exposure-relevant concentrations	Published iPSC-RPE and retinal NAM studies support lysosomal alkalinization and related mechanistic responses to CQ/HCQ (Achberger et al., 2019; Chandler et al., 2020; Farnoodian et al., 2022). Evidence currently supports mechanistic concordance; sensitivity, specificity, and prospective prediction across chemically diverse compounds remain unestablished
Barrier-disruption toxicity (Obert et al., 2017; Niu et al., 2023)	Stable polarized monolayer; reproducible baseline TEER; continuous ZO-1/occludin/claudin organization; low baseline paracellular permeability	TEER kinetics; tracer permeability; junctional-protein localization; morphology; recovery after washout	Sodium iodate and/or defined inflammatory/oxidative barrier stressors as assay-performance controls	Vehicle/untreated control; non-barrier-disrupting comparator under matched exposure conditions	For future qualification, baseline barrier measures and vehicle controls could be evaluated against laboratory-specific historical distributions. Positive controls would be expected to demonstrate a reproducible dynamic range and concentration-dependent barrier response. Candidate acceptance criteria could incorporate control performance, intra- and inter-run variability, followed by assessment of inter-laboratory reproducibility	TEER and junctional endpoints are established measures of epithelial barrier function, but retinal-toxicity-specific classification thresholds and prospective predictive performance have not been established
Mitochondrial/metabolic toxicity (Regent et al., 2019; Blasiak et al., 2022; Ghosh et al., 2024; Tsikandelova et al., 2024)	Stable basal respiration; adequate spare respiratory capacity; stable ATP production; low spontaneous oxidative stress	OCR/ECAR; ATP content; mitochondrial membrane potential (e.g., TMRM/JC-1); mitochondrial ROS; recovery following washout	Rotenone; antimycin A; oligomycin as pathway-performance controls	Vehicle/untreated control; compounds without expected mitochondrial liability at exposure-matched concentrations	Assays should demonstrate stable baseline bioenergetics and reproducible responses to pathway controls. Concentration-response relationships, dynamic range, repeatability, and recovery should be predefined. Mitochondrial changes should be interpreted together with general cytotoxicity to distinguish primary mitochondrial dysfunction from terminal cell injury	Mechanistically relevant and technically measurable in RPE models, but current evidence is insufficient to establish retinal-specific sensitivity, specificity, or prospective clinical predictivity
Ion/fluid-transport toxicity/MEKAR-like functional perturbation (Lozano et al., 2025; Lozano et al., 2026; Buenasmañanas-Maeso et al., 2022)	Mature polarized monolayer; preserved baseline TEER; appropriate AQP1 and ion/solute transporter expression; intact secretory/transport function	AQP1 and transporter expression/function; directional fluid transport; POS handling; lysosomal acidification where mechanistically relevant; TEER; washout/recovery kinetics	Selumetinib; additional clinically associated MEK inhibitors should be incorporated during future validation	Vehicle control; appropriately selected pharmacological/structural comparators without clinically meaningful MEKAR where available	Reference responses should be reproducible and exposure-dependent and should capture functional perturbation and reversibility rather than cell death alone. Washout/recovery should be prespecified. Future qualification requires testing across multiple MEK inhibitors and negative comparators with predefined decision thresholds and clinically relevant exposure ranges	Published iPSC-RPE studies support changes in POS handling, AQP1/transport-related pathways, and inter-individual susceptibility to selumetinib (Lozano et al., 2025; Lozano et al., 2026). Evidence supports mechanistic and phenotypic concordance, but available datasets are small and prospective predictivity remains unvalidated
Inflammatory/complement-associated RPE activation (Fields et al., 2020; Navneet and Rohrer, 2026)	Low basal inflammatory tone; preserved barrier function; reproducible inducibility of inflammatory signaling	NF-κB activation; NLRP3/caspase-1; IL-1β, IL-18, IL-6, MCP-1; complement-related endpoints; TEER/permeability where relevant	LPS/ATP or complement-active challenge as pathway-performance controls	Vehicle/untreated control; inactive serum/complement condition where appropriate	Basal inflammatory markers should remain within historical limits and positive controls should produce reproducible activation without excessive nonspecific cell death. Multiparametric interpretation is required because inflammatory readouts alone are not retinal-toxicity-specific	Useful for mechanistic pathway assessment, but monoculture iPSC-RPE does not capture immune-cell-dependent or full neurovascular inflammatory interactions. These endpoints are not independently predictive of retinal toxicity
Chronic anti-VEGF/long-term homeostatic perturbation (Marneros et al., 2005; Saint et al., 2009)	Long-term culture stability; preserved epithelial polarity and barrier function; reproducible polarized VEGF/PEDF secretion; preferably endothelial interaction for vascular COUs	Apical/basal VEGF and PEDF; TEER/permeability; mitochondrial function; endothelial response in co-culture or oBRB systems; inflammatory/complement signaling; recovery following treatment withdrawal	VEGF-neutralizing biologics as context-specific exposure compounds rather than established retinal-toxicity benchmarks	Vehicle/isotype control; untreated long-term culture; appropriate biologic control lacking VEGF-neutralizing activity where feasible	Qualification should emphasize chronic/repeated exposure, long-term vehicle stability, time-dependent functional changes, recovery, and endothelial–RPE interaction where relevant. No universal risk threshold should be assigned until clinically anchored exposure-response datasets are available	Exploratory/future COU. Biological plausibility exists, but iPSC-RPE-specific predictive validation and clinically anchored decision thresholds have not been established

The CQAs, controls, performance characteristics, and acceptance considerations summarized in this table represent a proposed framework for future COU-specific qualification rather than experimentally validated thresholds or established regulatory acceptance criteria. Literature-supported observations and the current evidence and predictive-validation status are summarized separately in the relevant columns. Unless otherwise indicated, no universal numerical thresholds have been validated for iPSC-RPE, retinal toxicity assays. Candidate acceptance ranges should be established for each laboratory and COU, from historical vehicle- and reference-control distributions and subsequently evaluated through intra-run, inter-run, and inter-laboratory validation, consistent with Good *In Vitro* Method Practices (OECD, 2025). For classification-oriented applications, independent positive and negative compound sets should be used to estimate sensitivity, specificity, positive predictive value, negative predictive value, and associated confidence intervals. Reference compounds should be selected according to the intended mechanism and COU; pathway-performance controls should not be interpreted as clinically validated retinal toxicants.

## 5 Conclusions and future perspectives

iPSC-RPE models have progressed from descriptive disease modeling systems to increasingly sophisticated human-relevant platforms for ocular safety assessment. Their translational value is derived not only from their structural similarity to native RPE but also from their ability to recapitulate functionally relevant biological processes that underlie drug-induced retinal toxicity, including epithelial polarity, barrier integrity, lysosomal degradation, photoreceptor outer segment phagocytosis, mitochondrial metabolism, and stress response signaling. Importantly, this review highlights that the potential utility of iPSC-RPE within a NAM framework is inherently context-dependent. Rather than serving as a universal replacement for existing *in vivo* or *in vitro* systems, iPSC-RPE platforms should be understood as modular, mechanism-specific tools whose potential utility depends on their alignment with a clearly specified COU. Within this framework, model suitability should be assessed according to the reproducibility, mechanistic fidelity, and decision relevance of defined functional outputs rather than by biological complexity alone.

A key conclusion emerging from this analysis is that disease modeling and toxicology should not be viewed as separate domains, but rather as mechanistically interconnected fields. iPSC-RPE disease models provide important evidence for human-relevant pathways, including lysosomal dysfunction, oxidative stress, lipid accumulation, complement dysregulation, and impaired epithelial transport. These pathways also contribute to the mechanistic basis of drug-induced retinal toxicity, suggesting that disease-derived phenotypes can be systematically repurposed to inform the selection and interpretation of toxicological endpoints. Despite these advances, several critical challenges remain before iPSC-RPE platforms can achieve the level of qualification and regulatory confidence needed for potential use within defined NAM applications. First, many current systems represent developmentally immature or early postnatal-like states rather than fully aged human RPE. This limits their ability to model age-dependent vulnerability, particularly in diseases such as age-related macular degeneration, where cumulative metabolic stress, mitochondrial decline, and chronic inflammatory activation play central roles. Although induced aging paradigms may reproduce selected features of aged RPE, no single approach currently recapitulates the multidimensional and cumulative nature of human RPE aging. Aging-related assay modifications should therefore be qualified according to the specific biological feature required by the intended COU. Second, inter-batch and inter-laboratory variability in differentiation protocols, maturation status, and functional performance remains a major barrier to reproducibility. Third, the lack of standardized criteria for defining model qualification and context-specific applicability remains a barrier to regulatory confidence. In addition, current monoculture-based iPSC-RPE systems cannot fully recapitulate the complexity of the *in vivo* retinal microenvironment. Key components such as Bruch’s membrane architecture, choroidal endothelial perfusion, immune cell interactions, and long-term extracellular matrix remodeling are not adequately represented in conventional models. As a result, certain classes of toxicity, particularly immune-mediated injury and chronic cumulative degeneration, remain difficult to predict using existing platforms. Future development of iPSC-RPE NAMs should therefore prioritize the establishment of a structured qualification roadmap. This includes the definition of consensus CQAs explicitly linked to specific COU, the development of standardized reference compound panels covering distinct toxicity mechanisms, and the implementation of cross-laboratory validation studies using harmonized protocols and shared performance benchmarks. Importantly, such efforts should focus not only on biological standardization but also on functional reproducibility and decision relevance. Emerging technologies are expected to play a critical role in this transition. Advanced systems such as oBRB organ-on-chip platforms, long-term perfusion-based culture systems, CRISPR-engineered disease or risk models, and AI-assisted phenotypic analysis offer opportunities to enhance physiological relevance and data integration. However, their adoption should be guided by demonstrated improvements in mechanistic interpretability, assay robustness, and predictive performance, rather than technological complexity alone. Finally, exposure-informed experimental design represents a key requirement for future NAM implementation. Incorporating clinically relevant concentration time profiles, drug accumulation dynamics, washout phases, and recovery responses will be essential for translating *in vitro* functional changes into meaningful safety decisions. This is particularly important for compounds with intracellular sequestration, long half-lives, or reversible pharmacological effects.

It should also be emphasized that RPE-centered NAMs address only part of the biological spectrum of drug-induced retinal toxicity. Compounds primarily affecting photoreceptors, retinal ganglion cells, Müller glia, vascular endothelial cells, or neurovascular interactions may require complementary human-relevant models based on these cell types. Retinal organoids, multicellular co-culture systems, and retina-/blood-retinal-barrier-on-chip platforms may therefore complement iPSC-RPE by capturing toxicities that depend on neuronal, glial, vascular, or intercellular mechanisms. Future ocular safety strategies will likely rely on a tiered NAM framework in which iPSC-RPE is deployed for RPE-relevant COU and is integrated with other retinal models when the anticipated mechanism of toxicity extends beyond the RPE.

In conclusion, iPSC-RPE should be viewed as an evolving, COU-defined experimental platform with potential future utility in mechanism-based ocular toxicity assessment. Broader implementation will require standardized CQAs, exposure-relevant experimental designs, inter-laboratory reproducibility, and clinically relevant validation datasets. With these advances, iPSC-RPE could become an important component of human-relevant ocular toxicology frameworks. Such models may complement existing preclinical approaches by improving mechanistic interpretability and supporting efforts to reduce reliance on animal testing.

Overall, current evidence supports mechanistic concordance more strongly than prospective predictivity. Establishing predictive performance will require blinded reference-compound studies and inter-laboratory validation within clearly defined COUs.
